# Effect of fibroblast growth factor 9 on the osteogenic differentiation of bone marrow stromal stem cells and dental pulp stem cells

**DOI:** 10.3892/mmr.2014.2998

**Published:** 2014-11-26

**Authors:** JINGTING LU, JIEWEN DAI, XUDONG WANG, MAOLIN ZHANG, PENG ZHANG, HAO SUN, XIULI ZHANG, HONGBO YU, WENBIN ZHANG, LEI ZHANG, XINQUAN JIANG, STEVE GUOFANG SHEN

**Affiliations:** 1 Department of Oral and Craniomaxillofacial Science, Shanghai Ninth People’s Hospital College of Stomatology, Shanghai Jiao Tong University School of Medicine, Shanghai 200011, P.R. China; 2 Oral Bioengineering Lab, Shanghai Key Laboratory of Stomatology, Shanghai Research Institute of Stomatology, Shanghai Ninth People’s Hospital Affiliated to Shanghai Jiao Tong University School of Medicine, Shanghai 200011, P.R. China

**Keywords:** fibroblast growth factor 9, osteogenic differentiation, bone marrow stromal stem cells, dental pulp stem cells

## Abstract

The role of fibroblast growth factor 9 (FGF9) in bone formation may depend on gene dosage, developmental stage, cell type or interactions with other cytokines. In the present study bone marrow stromal stem cells (BMSCs) and dental pulp stem cells (DPSCs) were cultured and osteogenically induced *in vitro*, treated with exogenous FGF9 at varying concentrations. Alkaline phosphatase staining, alizarin red S staining, reverse transcription quantitative polymerase chain reaction and western blot analyses were performed in order to investigate the gene expression levels of osteogenic markers. The results of the present study demonstrated that FGF9 enhanced the phosphorylation of extracellular signal-regulated kinase 1/2 (ERK1/2) during osteogenic induction in BMSCs and DPSCs, which are derived from different tissues*.* FGF9 also inhibited the osteogenic differentiation of BMSCs and DPSCs through the activation of ERK1/2. These findings suggested that FGF9 may be an inhibitor of osteogenesis in mesenchymal stem cells *in vitro* and its application *in vivo* requires investigation in the future.

## Introduction

Fibroblast growth factor 9 (FGF9) is important in a variety of biological processes, including the development of the lung, limb, testis and skeletal system in vertebrates (1–7). In skeletal development, FGF9 may have complex and important roles in endochondral ossification and intramembranous bone formation (4,5,7). Within the context of long bone development, Hung *et al* (4) demonstrated that FGF9 promotes chondrocyte hypertrophy in the early stages of skeletal development and regulated the vascularization of the growth plate and osteogenesis in the later stages. In addition, mice lacking FGF9 exhibited decreased chondrocyte proliferation and delayed onset of chondrocyte hypertrophy defects in skeletal vascularization, which led to abnormal osteogenesis. Behr *et al* (7) revealed that FGF9 promoted bone healing by initiating angiogenesis through vascular endothelial growth factor (VEGF)-α, which further supported the concept that the healing processes observed in adults may be a recapitulation of those observed during embryonic skeletal development. Ignelzi *et al* (8) demonstrated that exogenous FGF9 leads to increased expression of Msh homeobox 2, followed by osteogenic differentiation induced by the sutural mesenchyme during craniofacial skeletal development in mouse calvaria. Furthermore, Govindarajan and Overbeek (5) revealed that the differentiation of cranial mesenchymal cells from the mesoderm, but not cranial neural crest cells, can be altered by FGF9 from intramembranous to endochondral ossification. Fakhry *et al* (9) also demonstrated that in cell populations comprising mature osteoblasts, proliferation is stimulated by FGF9; however, this does not occur in populations of undifferentiated precursor cells. Although these findings collectively suggested that FGF9 is able to promote bone formation, other studies have demonstrated contradictory results. Weksler *et al* (10) indicated that FGF9 weakly promoted the proliferation of a rat calvaria-derived cell line *in vitro,* but inhibited its terminal differentiation. Garofalo *et al* (11) observed that, in transgenic mice, the proliferation and terminal differentiation of chondrocytes in the cartilage was inhibited following induced overexpression of FGF9. In addition, FGF9-FGFR3 signaling physiologically inhibited endochondral ossification, leading to a phenotype which is characteristic of skeletal dysplasia (11). Wu *et al* (12) demonstrated that a loss-of-function mutation in FGF9 led to the proliferation and differentiation of chondrocytes, with an increase in osteogenic differentiation and matrix mineralization in bone marrow-derived mesenchymal stem cells (BMSCs) and joint ankylosis. Additionally, a previous study by our group revealed that, in dental pulp stem cells (DPSCs), exogenous FGF9 led to the promotion of chondrogenesis and partial inhibition of mineralization (13).

Although previous studies have revealed that FGF9 is important in skeletal development, their apparently contradictory results also indicated that the action of FGF9 is complex and that the biological effects of FGF9 may depend on the gene dosage, developmental stage, cell type and interactions with other cytokines. In addition, the activities of genes downstream of FGF9, including FGF receptor 3 and mitogen-activated protein-kinase (MAPK), which include extracellular signal-regulated kinase (ERK), p38 and c-Jun N-terminal kinase (JNK), may also affect its function (12–14). However, the different types of MAPK are activated by different extracellular stimuli and differ in their downstream targets, therefore having distinct roles in cellular responses. In the present study, the effects of FGF9 on osteogenic differentiation in BMSCs and DPSCs were compared to investigate whether FGF9 differs in its osteogenic induction capabilities in different cells. In addition, the present study examined whether ERK1/2, a gene downstream of FGF9, differs in its role in osteogenesis in different types of cell.

## Materials and methods

### Culture of BMSCs and DPSCs

The method of the present study was reviewed and approved by the Ethical Committee of Shanghai Ninth People’s Hospital, Shanghai Jiao Tong University School of Medicine (Shanghai, China). DPSCs were isolated using direct cell outgrowth from pulp tissue explants and cultured as previously reported (13). Briefly, healthy human third molars were collected from adults aged between 16 and 25 years (mean, 18±3.2) at the Oral and Maxillary Facial Surgery Clinic of The Ninth People’s Hospital, Shanghai Jiao Tong University School of Medicine. All individuals provided informed consent. Following cleaning of the teeth, the pulp chambers were accessed by cutting the cementoenamel junction with a sterile fissure dental bur. Following exposure of the pulp, the pulp tissue was removed in fragments of ~0.5 mm, which were then placed onto a 6-cm culture dish containing Dulbecco’s modified Eagle’s medium (DMEM; Gibco-BRL, Grand Island, NY, USA) supplemented with 20% fetal bovine serum (FBS; Hyclone, Logan, UT, USA) and antibiotics (Gibco-BRL) prior to incubation at 37°C in 5% CO_2_. At confluence, the outgrown cells were transferred onto a 10-cm dish and then continuously passaged for further experiments. All experiments were performed with mixed cells from multiple patients and cells from the third passage were used.

Four-week-old male Sprague Dawley rats were obtained from the Ninth People’s Hospital Animal Center (Shanghai, China) and the bone marrow was extracted from the femurs and tibias, as described previously (15). All procedures were approved by the Animal Research Committee of the Shanghai Ninth People’s Hospital. The BMSCs were cultured in DMEM supplemented with 10% FBS and 1% penicillin/streptomycin at 37°C in 5% CO_2_ for 5–7 days. When they had reached 80–90% confluence, the BMSCs were transferred onto a 10-cm dish and then continuously passaged for further experiments. The culture medium was replaced every three days and the BMSCs at passages two to four were used in subsequent experiments.

### Osteogenic induction (OI)

The osteogenic medium used for the differentiation of the cultured BMSCs and DPSCs comprised DMEM, 10% FBS, 10^−8^ M dexamethasone, 5 mmol/l KH_2_PO_4_, 50 mg/ml L-ascorbic acid and 50 mg/ml gentamycin (Sigma-Aldrich, St. Louis, MO, USA). The medium was replaced every 2–3 days for 14–28 days. The conditions used in the different experiments were as follows: BMSCs/DPSCs cultured in DMEM with 10% FBS (control; CON group); BMSCs/DPSCs cultured in the OI medium described above (OI group); BMSCs/DPSCs cultured in OI medium with 20 ng/ml FGF9 protein (OI+FGF9 group) and BMSCs/DPSCs/CSCs cultured in OI medium with 20 ng/ml FGF9 protein, U0126, SB203580 and SP600125 (OI+FGF9+U/SB/SP group). The inhibitors U0126, SB203580 and SP600125 at concentrations of 10, 10 and 25 μM, respectively, were added to the DMEM medium 2 h prior to its replacement with OI medium, which also contained these three inhibitors, for continuous culture.

### MTT assay

A total of 10^4^ BMSCs/well were seeded into 96-well plates and subsequently cultured in 200 μl DMEM with 10% FBS and exogenous FGF9 protein at different concentrations (1, 5, 10, 20 or 40 ng/ml). The proliferation of the BMSCs was then evaluated at different time-points (1, 3, 5, 7 and 9 days) using an MTT assay. Briefly, the BMSCs were incubated with 0.5 mg/ml MTT (Sigma-Aldrich) under normal culture conditions for another 4 h. The medium was then removed, 200 μl DMSO (Sigma-Aldrich) was added to each well and the plates were agitated for 10 min. The absorbance of the solution was then measured at 490 nm and corrected with an NP blank using a spectrophotometer (Elx 800; BioTek Instruments, Inc., Winooski, VT, USA). A growth curve of the BMSCs was then generated based on the absorbance values measured at different time-points.

### Alkaline phosphatase (ALP) staining and alizarin red staining

ALP staining of the BMSCs and DPSCs *in vitro* was performed 7 days after treatment using an ALP staining kit (Biyuntian Biotech Co., Ltd., Shanghai, China). Briefly, the cultured cells were washed twice using phosphate-buffered saline (PBS; Gibco-BRL), fixed in 4% paraformaldehyde and stained using an ALP staining kit for 30 min. This was followed by thorough washing and images were then captured with a digital single lens reflex camera (Olympus, Osaka, Japan). For the alizarin red S staining, the cultured cells were fixed in 95% ethanol 21 days after treatment. The cells were then stained using 1% Alizarin Red S (Sigma-Aldrich) for 20 min. Subsequently, the cell samples were thoroughly washed and images were captured.

### Reverse transcription quantitative polymerase chain reaction (RT-qPCR)

Total cellular RNA was extracted from different cell samples using TRIzol reagent (Invitrogen Life Technologies, Carlsbad, CA, USA) according to the manufacturer’s instructions. The total RNA was converted to cDNA using a Prime Script-RT reagent kit (Takara Bio Inc., Shiga, Japan) according to the manufacturer’s instructions. The gene-specific primers used for the amplification of collagen type 1 (COL1), runt-related transcription factor 2 (Runx2), osteopontin (OPN) and osteocalcin (OCN) are listed in Table I. All RT-qPCR reactions were performed using a SYBR Green system according to the manufacturer’s instructions with 20 μl reaction volumes in 96-well microwell plates and using a MyiQ Real-Time PCR Detection system (Bio-Rad, Hercules, CA, USA) (13). Data analysis was based on calculating the relative expression levels of these genes compared with the controls.

### Western blot analysis

The BMSCs and DPSCs were collected and washed three times using ice-cold PBS. The samples were then lysed using radioimmunoprecipitation assay buffer containing protease inhibitors (Biyuntian Biotech Co., Ltd.). Subsequently, the lysates were centrifuged and the supernatants were boiled in SDS sample buffer (Sigma-Aldrich). The samples were then separated by 10% SDS-PAGE and transferred onto a polyvinylidene difluoride membrane (Bio-Rad Laboratories, Inc., Hercules, CA, USA). The membranes were inhibited using 5% milk for 2 h and then incubated with rabbit anti-mouse ERK1/2, p-ERK1/2, p38, phospho (p)-p38 or tubulin antibodies (1:1,000 dilutions; Cell Signaling Technology Inc., Danvers, MA, USA) or goat anti-mouse Runx2 or COL1 antibodies (1:1,000; R&D Systems, Minneapolis, MN, USA) in PBS with Tween-20 buffer overnight. Finally, the membranes were incubated with horseradish peroxidase-conjugated secondary antibody (1:3,000, Santa Cruz Biotechnology, Inc., Santa Cruz, CA, USA) and visualized using an enhanced chemiluminescence western blotting kit (Pierce Biotechnology, Inc., Rockford, IL, USA).

### Statistical analysis

The data are expressed as the mean ± standard deviation. Data analysis was performed using an independent samples test with SPSS 18.0 software (International Business Machines, Armonk, NY, USA). P<0.05 was considered to indicate a statistically significant difference.

## Results

### Effect of exogenous FGF9 on the proliferation of BMSCs in vitro

**Figure 1:**
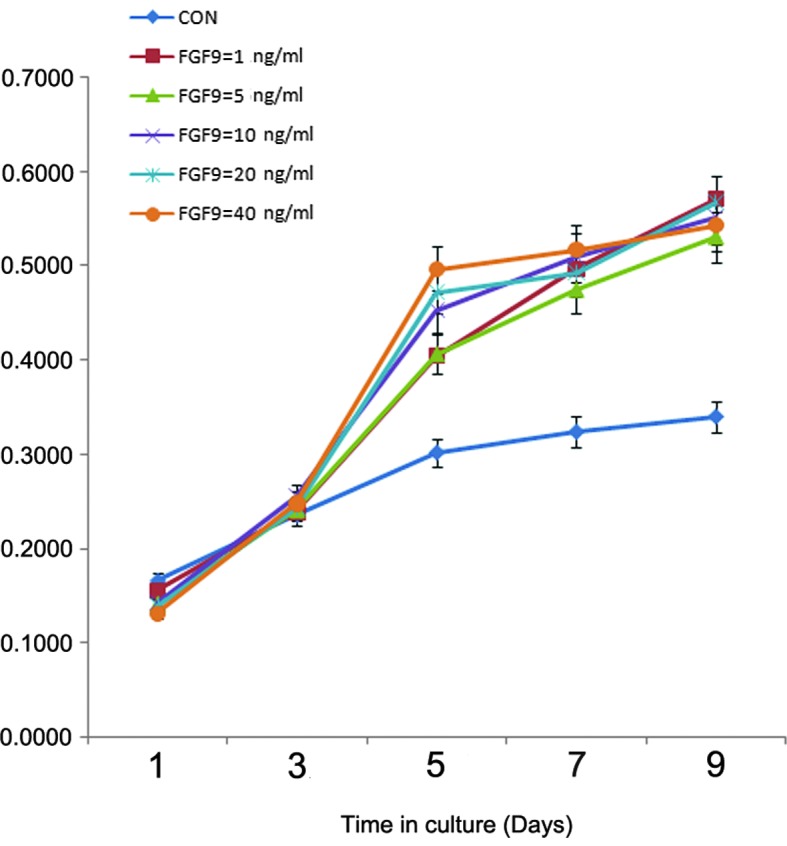
Effect of exogenous FGF9 on BMSC proliferation. The effects of different concentrations of exogenous FGF9 on the proliferation of BMSCs were examined using an MTT assay on days 1, 3, 5, 7 and 9. BMSCs were cultured in Dulbecco’s modified Eagle’s medium as a control. The FGF9-treated cells exhibited a several-fold increase in cell proliferation during the first three days of culture. Statistically significant differences were detected between the control and FGF9-treated cells, whereas no statistically significant differences were detected among the treated cells with different concentrations of FGF9 (1, 5, 10, 20 and 40 ng/ml). Data are expressed as the mean ± standard deviation of three independent experiments. BMSC, bone marrow stromal stem cell; Con, control; FGF9, fibroblast growth factor 9.

The effects of exogenous FGF9 on BMSC viability and proliferation were examined using an MTT assay, with BMSCs cultured in regular DMEM medium as a control. The FGF9-treated cells exhibited a several-fold increase in cell number during the first three days of culture. Subsequently, the control cells demonstrated a slight increase in cell number, whereas a substantial increase in cell number was observed in the cells treated with FGF9 between days three and nine. However, no significant differences were observed in proliferation among the cells treated with exogenous FGF9 at different concentrations (1, 5, 10, 20 and 40 ng/ml; Fig. 1). Therefore, the median concentration of 20 ng/ml was used in the subsequent experiments (Fig. 1).

### Effect of exogenous FGF9 on the osteogenic differentiation of BMSCs in vitro

**Figure 2:**
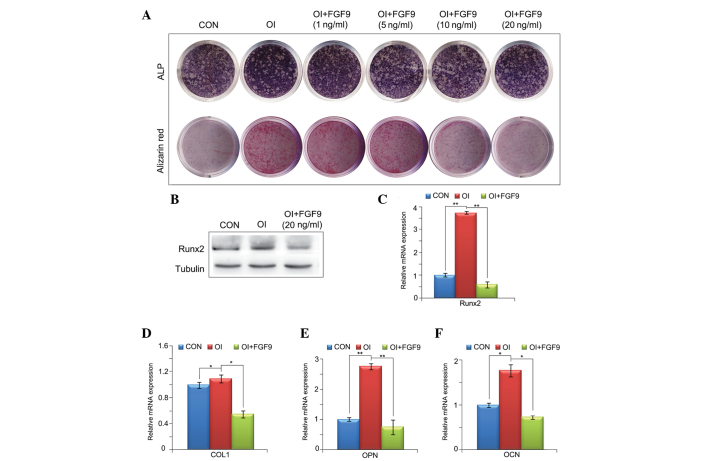
Effect of exogenous FGF9 on the osteogenic differentiation of BMSCs *in vitro*. (A) ALP staining of cultured monolayer BMSCs at day 7 and alizarin red staining at day 21. FGF9 inhibited the ALP expression and matrix mineralization of BMSCs in a concentration-dependent manner. (B) Effects of FGF-9 on Runx2 protein levels in BMSCs 12 h after treatment were investigated by western blot analysis. (C–F) mRNA expression levels of four osteogenic transcription factors, Runx2, COL1, OPN and OCN, at different stages of osteogenesis in the FGF9-treated (20 ng/ml) BMSCs were investigated using reverse transcription quantitative polymerase chain reaction (12 h and 7, 14 and 21 days after treatment, respectively). The gene and protein expression levels of these four osteogenic transcription factors were reduced by exogenous FGF9. Data are expressed as the mean ± standard deviation of three independent experiments. ^*^P<0.05, ^**^P<0.01. BMSCs, bone marrow stromal stem cells; ALP, alkaline phosphatase; Runx2, runt-related transcription factor 2; COL1, collagen type 1; OPN, osteopontin; OCN, osteocalcin; CON, Dulbecco’s modified Eagle’s medium; OI, osteogenic induction medium; FGF9, fibroblast growth factor 9.

ALP staining, RT-qPCR and western blot analysis were performed to investigate the effect of exogenous FGF9 on the osteogenic differentiation of BMSCs *in vitro*. As shown in Fig. 2A, the ALP and alizarin red staining demonstrated that FGF9 inhibited the osteogenic differentiation of BMSCs in a concentration-dependent manner. The RT-qPCR and western blot analyses revealed that the gene expression levels of four osteogenic markers, Runx2, COL1, OPN and OCN, were reduced by treatment with 20 ng/ml exogenous FGF9 for 12 h and 7, 14 and 21 days. This further confirmed that exogenous FGF9 impaired the osteogenic capacity of BMSCs (Fig. 2B–F).

### Effect of exogenous FGF9 on the phosphorylation of ERK1/2, p38 and JNK in BMSCs in vitro

**Figure 3:**
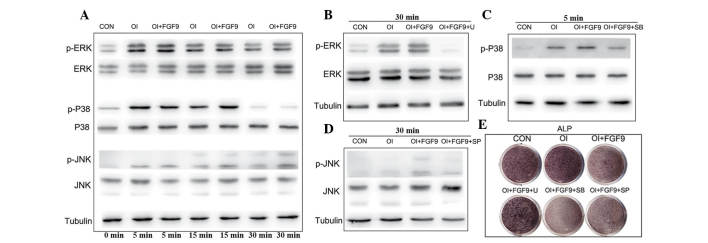
Effect of the FGF9/mitogen-activated protein kinase signaling pathway in the osteogenic differentiation of BMSCs *in vitro*. (A) ERK1/2, p-ERK1/2, p38, p-p38, JNK and p-JNK protein levels in FGF9-treated (20 ng/ml) BMSCs were measured by western blot analysis at the indicated time-points. Tubulin was used to demonstrate equal loading of all samples. FGF9 significantly enhanced the phosphorylation of ERK1/2, P38 and JNK in BMSCs compared with that in the CON and OI groups. (B–D) Cultured BMSCs were pretreated with the protein synthesis inhibitors, ERK1/2 inhibitor U0126 (10 μM), p38 inhibitor SB203580 (10 μM) and JNK inhibitor SP600125 (25 μM), 2 h prior to experiments. Western blot analysis revealed that the protein synthesis inhibitor inhibited phosphorylation of ERK1/2, p38 and JNK at the indicated time-points. (E) ALP staining of the FGF9/inhibitor-treated monolayer BMSCs. After 7 days of osteogenic culture induction, ALP expression in the FGF9-treated BMSCs was reduced and this was rescued by the treatment with ERK1/2 inhibitor. However, rescue was not observed in the BMCs treated with the p38 and JNK inhibitors. The results are representative of three independent experiments. BMSC, bone marrow stromal stem cell; p-ERK1/2, phosphorylated extracellular signal-regulated kinase 1/2; JNK, c-Jun N-terminal kinase; CON, Dulbecco’s modified Eagle’s medium; OI, osteogenic induction medium; FGF9, fibroblast growth factor 9; U, 10 μM U0126; SB, 10 μM SB203580; SP, 25 μM SP600125; ALP, alkaline phosphatase.

Previous studies have revealed that the MAPK signaling pathway is important in the osteogenic differentiation of mesenchymal stem cells. The results of the western blot analysis in the present study revealed that p38, ERK1/2 and JNK phosphorylation were augmented in the BMSCs cultured in the osteogenic medium compared with the controls (Fig. 3A). Furthermore, the incubation of BMSCs cultured in osteogenic medium containing 20 ng/ml FGF9 significantly enhanced MAPK signaling, leading to increased levels of phosphorylated ERK1/2, p38 and JNK, without altering the total quantities of these proteins. The increased levels of phosphorylated ERK1/2 were first observed at 5 min and peaked 5 min after FGF9 treatment, maintaining a high level thereafter. The level of phosphorylated p38 also increased and peaked at 15 min prior to gradually reducing. Similarly, phosphorylated JNK increased and peaked at 30 min (Fig. 3A).

### Effects of the FGF9/MAPK signaling pathway in the osteogenic differentiation of BMSCs in vitro

**Figure 4:**
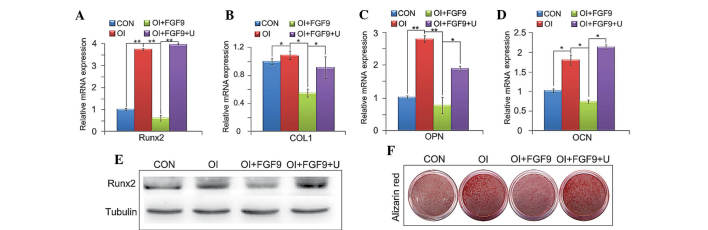
Effect of the FGF9/ERK1 signaling pathway on the osteogenic differentiation of BMSCs *in vitro*. (A–D) mRNA expression of four osteogenic marker genes, Runx2, COL1, OPN and OCN, in treated BMSCs were investigated using reverse transcription quantitative polymerase chain reaction. The gene expression levels of the four osteogenic transcription factors were downregulated by exogenous FGF9 (20 ng/ml) after 12 h and 7, 14 and 21 days after treatment, respectively. U0126 (10 μM) reversed the inhibited expression of the four osteogenic marker genes. (E) Levels of Runx2 protein expression in BMSCs were investigated by western blot analysis. (F) Alizarin red staining of treated monolayer BMSCs. Western blot analysis of Runx2 and alizarin red staining confirmed that the inhibition of the osteogenic capacity of BMSCs by FGF9 was reversed when ERK1/2 phosphorylation was inhibited. Values are expressed as the mean ± standard deviation of triplicate independent experiments.^*^P<0.05, ^**^P<0.01. CON, Dulbecco’s modified Eagle’s medium; OI, osteogenic induction medium; FGF9, 20 ng/ml exogenous fibroblast growth factor 9; U, 10 μM U0126; Runx2, runt-related transcription factor 2; BMSCs, bone marrow stromal stem cells; COL1, collagen type 1; OPN, osteopontin; OCN, osteocalcin.

A previous study by our group demonstrated that FGF9 promotes chondrogenesis and, at the same time, inhibits the hypotrophy in DPSCs by binding to FGFR3 and enhancing ERK1/2 phosphorylation (13). To investigate the role of the MAPK pathway in the osteogenic differentiation of BMSCs, the drugs U0126 (10 μM), SB203580 (10 μM) and SP600125 (25 μM), which inhibit the phosphorylation of ERK1/2, p38 and JNK, respectively, were applied and their efficacy was confirmed by western blot analysis (Fig. 3B–D). Subsequent ALP staining revealed that exogenous FGF9 inhibited the expression of ALP, whereas exogenous FGF9 combined with the inhibition of p38 and JNK phosphorylation had an apparent synergistic effect on the inhibition of ALP expression in BMSCs (Fig. 3E). By contrast, the inhibition of ERK1/2 phosphorylation reversed the effect of FGF9 and increased the expression of ALP (Fig. 3E). RT-qPCR and western blot analyses further demonstrated that FGF9 treatment decreased the expression of the osteogenic genes COL1 and Runx2, whereas U0126 reversed the inhibition of the expression of Runx2, COL1, OPN and OCN (Fig. 4A–D). Western blot analysis of Runx2 and alizarin red staining further confirmed that inhibition of the osteogenic capacity of BMSCs by FGF9 was reversed when ERK1/2 phosphorylation was inhibited (Fig. 4E and F).

### Effects of the FGF9/ERK1/2 signaling pathway in the osteogenic differentiation of DPSCs in vitro

**Figure 5:**
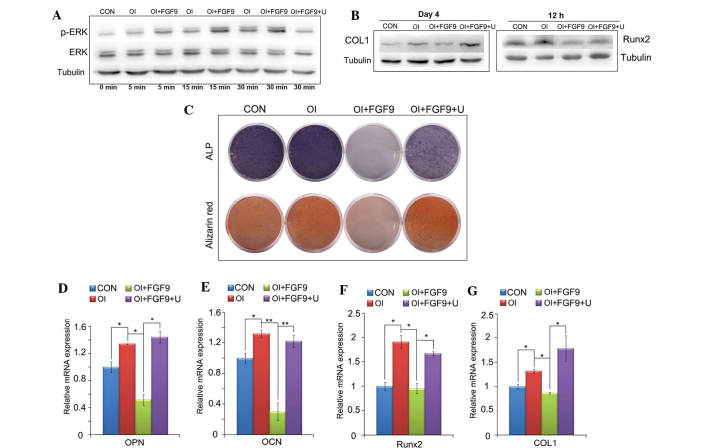
Effect of the FGF9/ERK signaling pathway on the osteogenic differentiation of DPSCs *in vitro*. (A) Expression of ERK1/2 and p-ERK1/2 in treated monolayer DPSCs was detected by western blot analysis at the indicated time-points. Tubulin was used to demonstrate equal loading of all samples. FGF9 (20 ng/ml) significantly enhanced the phosphorylation of ERK1/2 in DPSCs compared with that in the CON and OI groups. (B) Protein levels of Runx2/COL1 12 h and 4 days after treatment in DPSCs were investigated by western blot analysis. (C) ALP staining at day 7 and alizarin red staining at day 21 of the FGF9/inhibitor-treated monolayer DPSCs. Western blot analysis for Runx2 and COL1, ALP and alizarin red staining revealed that the inhibition of the osteogenic capacity of DPSCs by FGF9 was reversed when ERK1/2 phosphorylation was inhibited. (D–G) mRNA expression levels of Runx2, COL1, OPN and OCN in treated DPSCs were investigated using reverse transcription quantitative polymerase chain reaction. The gene and protein expression levels of these four osteogenic transcription factors were downregulated by exogenous FGF9 (20 ng/ml) 12 h and 7, 14 and 21 days after treatment, respectively. U0126 (10 μM) reversed the inhibited expression of the four osteogenic marker genes. Data are expressed as the mean ± standard deviation of triplicate independent experiments. ^*^P<0.05, ^**^P<0.01. DPSCs, dental pulp stem cells; CON, Dulbecco’s modified Eagle’s medium; OI, osteogenic induction medium, FGF9, 20 ng/ml exogenous fibroblast growth factor 9; U, 10 μM U0126; ERK, extracellular signal-regulated kinase; p-ERK, phosphorylated ERK; Runx2, runt-related transcription factor 2; COL1, collagen type 1; OPN, osteopontin; OCN, osteocalcin; ALP, alanine phosphatase.

In the present study, the results of western blot analysis demonstrated that the phosphorylation of ERK1/2 was augmented in DPSCs cultured in osteogenic medium. The results also revealed that the incubation of DPSCs cultured in osteogenic medium including 20 ng/ml FGF9 markedly augmented the phosphorylation of ERK1/2, beginning at 5 min and gradually increasing thereafter, whereas 10 μM U0126 effectively inhibited the phosphorylation of ERK1/2 (Fig. 5A). Similar to the results observed in the BMSCs, western blot analysis demonstrated that FGF9 inhibited the expression of COL1 and Runx2 in the DPSCs, whereas the inhibition of ERK1/2 phosphorylation reversed the inhibitory effect of FGF9 (Fig. 5B). The results of the ALP and alizarin red staining also indicated that FGF9 inhibited the expression of ALP and ossification, which was reversed when combined with the inhibition of ERK1/2 phosphorylation (Fig. 5C). The RT-qPCR analysis further revealed that FGF9 treatment decreased the expression of Runx2, COL1, OPN and OCN, whereas U0126 reversed this inhibitory effect and upregulated the expression of Runx2, COL1, OPN and OCN (Fig. 5D–G).

## Discussion

The present study provided evidence that the FGF9-ERK1/2 signaling pathway is important in the osteogenic differentiation process in different cell types. The results revealed that FGF9 inhibited the osteogenic differentiation of BMSCs and DPSCs *in vitro*, contradicting previous findings suggesting that FGF9 is necessary for growth plate development, osteogenesis and long bone repair *in vivo* (4,7). These differences may be attributed to the fact that the BMSCs in the present *in vitro* study were directly induced to differentiate into osteoblasts, whereas previous *in vivo* studies focused mainly on endochondral ossification, in which mesenchymal stem cells first differentiate into chondrocytes and are then gradually replaced by bone tissue. Previous studies also demonstrated that FGF9 promotes the differentiation and chondrogenesis of stem cells. Weksler *et al* (10) observed that FGF9 led to the promotion of proliferation of an *in vitro* rat calvaria-derived cell line; however, it caused inhibition of its terminal differentiation. Previous findings by our group revealed that FGF9 promotes the chondrogenesis and simultaneously inhibits the hypertrophy of DPSCs (13). Thus, although FGF9 is able to impair the osteogenic differentiation of stem cells and inhibit the hypertrophy of chondrocytes, endochondral ossification remains enhanced due to the increased cartilage formation. Additionally, a number of other factors, including bone morphogenetic proteins-2 and 4, promote osteoblast differentiation and may compensate for the inhibitory effect of FGF9 on osteogenic differentiation. Several previous studies have also demonstrated that FGF9 may promote angiogenesis through the expression of VEGF, thereby promoting bone formation (4,7,16). Mice lacking FGF9 have been observed to exhibit reduced chondrocyte proliferation, delayed chondrocyte hypertrophy and defects in skeletal vascularization leading to abnormalities in osteogenesis. These findings suggest that FGF9 regulates skeletal development *in vivo* at different stages and that the biological effect of FGF9 *in vivo* may depend on gene dosage, spatial and temporal expression patterns, developmental stage or interaction with other cytokines. In the present *in vitro* study, FGF9 had an inhibitory effect on the osteogenic differentiation of BMSCs and DPSCs. Combined with previous findings by our group that FGF9 simultaneously promotes the chondrogenesis and inhibits the hypertrophy in DPSCs, this suggested that FGF9 may be applied to promote the chondrogenesis of mesenchymal stem cells whilst inhibiting osteogenesis *in vitro*. As BMSCs originate from mesoderm, whereas DPSCs may be derived from neural ectoderm (17), the results of the present study indicated that the effects of FGF9 on osteogenic differentiation are similar in stem cells originating from the mesoderm or ectoderm *in vitro*. Therefore, potential methods for the application of FGF9 to promote chondrogenesis *in vivo* require investigation in the future.

Previous studies have revealed that the FGF9/FGFR3 signaling pathway activates the ERK1/2 pathway to regulate the chondrogenic or osteogenic differentiation of BMSCs (12). Several previous studies have also indicated a complex role for the ERK1/2 signaling pathway during chondrogenesis and osteogenesis in mesenchymal stem cells (MSCs) (18). Certain studies have demonstrated that activation of the ERK/MAPK signaling pathway promotes chondrogenesis and inhibits hypertrophy in MSCs, whereas others have demonstrated that inhibition of the ERK/MAPK signaling pathway promotes osteogenesis in MSCs (19,20). In the present study, FGF9 enhanced the phosphorylation and activation of ERK1/2 in BMSCs and DPSCs to inhibit their osteogenic differentiation. These findings revealed that FGF9 activated ERK1/2, which was followed by the inhibition of osteogenic differentiation in BMSCs and DPSCs. These findings were consistent with those of previous studies, in which decreased phosphorylation of ERK1/2 promoted the osteogenic differentiation of BMSCs, whereas enhanced ERK1/2 phosphorylation promoted the osteogenic differentiation of cranial suture cells, leading to craniosynostosis (21,22).

In conclusion, the present study revealed that FGF9 enhanced the phosphorylation of ERK1/2 in BMSCs and DPSCs during osteogenic induction *in vitro* and inhibited the osteogenic differentiation of BMSCs and DPSCs through the activation of ERK1/2. These findings suggested that FGF9 may be an inhibitor of osteogenesis in MSCs *in vitro*. However, the *in vivo* application of this interaction requires further investigation.

## Figures and Tables

**Table I:** Primer sequences for reverse transcription quantitative polymerase chain reaction.

Gene name	Primer sequence
Rat GAPDH	F, 5′-CCTGCACCACCAACTGCTTA-3′ R, 5′-GGCCATCCACAGTCTTCTGAG-3′
Rat Runx2	F, 5′-TCTCTGACCGCCTCAGTGATT-3′ R, 5′-TGTGTCTGCCTGGGATCTGTA-3′
Rat COL1	F, 5′-CTGCCCAGAAGAATATGTATCACC-3′ R, 5′-GAAGCAAAGTTTCCTCCAAGACC-3′
Rat OPN	F, 5′-GGAGTCCGATGAGGCTATCAA-3′ R, 5′-TCCGACTGCTCAGTGCTCTC-3′
Rat OCN	F, 5′-GCCCTGACTGCATTCTGCCTCT-3′ R, 5′-TCACCACCTTACTGCCCTCCTG-3′
Human GAPDH	F, 5′-TTCGACAGTCAGCCGCATCTT-3′ R, 5′-ATCCGTTGACTCCGACCTTCA-3′
Human Runx2	F, 5′-GCCTTCAAGGTGGTAGCCC-3′ R, 5′-CGTTACCCGCTATGACAGTA-3′
Human COL1	F, 5′-TCCAACGAGATCGAGATCC-3′ R, 5′-AAGCCGAATTCCTGGTCT-3′
Human OPN	F, 5′-CATGAGAATTGCAGTGTTTGCT-3′ R, 5′-CTTGCAAGGGTCTGTGGGG-3′
Human OCN	F, 5′-CCCCCTCTAGCCTAGGACC-3′ R, 5′-ACCAGGTAATGCCAGTTTGC-3′

F, forward; R, reverse; Runx2, runt-related transcription factor 2; COL1, collagen type 1; OPN, osteopontin; OCN, osteocalcin.

## References

[1] Sylvestersen KB, Herrera PL, Serup P, Rescan C (2011). Fgf9 signalling stimulates Spred and Sprouty expression in embryonic mouse pancreas mesenchyme. Gene Expr Patterns.

[2] Geske MJ, Zhang X, Patel KK, Ornitz DM, Stappenbeck TS (2008). Fgf9 signaling regulates small intestinal elongation and mesenchymal development. Development.

[3] White AC, Lavine KJ, Ornitz DM (2007). FGF9 and SHH regulate mesenchymal Vegfa expression and development of the pulmonary capillary network. Development.

[4] Hung IH, Yu K, Lavine KJ, Ornitz DM (2007). FGF9 regulates early hypertrophic chondrocyte differentiation and skeletal vascularization in the developing stylopod. Dev Biol.

[5] Govindarajan V, Overbeek PA (2006). FGF9 can induce endochondral ossification in cranial mesenchyme. BMC Dev Biol.

[6] DiNapoli L, Batchvarov J, Capel B (2006). FGF9 promotes survival of germ cells in the fetal testis. Development.

[7] Behr B, Leucht P, Longaker MT, Quarto N (2010). Fgf-9 is required for angiogenesis and osteogenesis in long bone repair. Proc Natl Acad Sci USA.

[8] Ignelzi MA, Wang W, Young AT (2003). Fibroblast growth factors lead to increased Msx2 expression and fusion in calvarial sutures. J Bone Miner Res.

[9] Fakhry A, Ratisoontorn C, Vedhachalam C (2005). Effects of FGF-2/-9 in calvarial bone cell cultures: differentiation stage-dependent mitogenic effect, inverse regulation of BMP-2 and noggin, and enhancement of osteogenic potential. Bone.

[10] Weksler NB, Lunstrum GP, Reid ES, Horton WA (1999). Differential effects of fibroblast growth factor (FGF) 9 and FGF2 on proliferation, differentiation and terminal differentiation of chondrocytic cells in vitro. Biochem J.

[11] Garofalo S, Kliger-Spatz M, Cooke JL (1999). Skeletal dysplasia and defective chondrocyte differentiation by targeted overexpression of fibroblast growth factor 9 in transgenic mice. J Bone Miner Res.

[12] Wu XL, Gu MM, Huang L (2009). Multiple synostoses syndrome is due to a missense mutation in exon 2 of FGF9 gene. Am J Hum Genet.

[13] Dai J, Wang J, Lu J (2012). The effect of co-culturing costal chondrocytes and dental pulp stem cells combined with exogenous FGF9 protein on chondrogenesis and ossification in engineered cartilage. Biomaterials.

[14] Ornitz DM, Marie PJ (2002). FGF signaling pathways in endochondral and intramembranous bone development and human genetic disease. Genes Dev.

[15] Zou D, Zhang Z, He J (2011). Repairing critical-sized calvarial defects with BMSCs modified by a constitutively active form of hypoxia-inducible factor-1alpha and a phosphate cement scaffold. Biomaterials.

[16] Frontini MJ, Nong Z, Gros R (2011). Fibroblast growth factor 9 delivery during angiogenesis produces durable, vasoresponsive microvessels wrapped by smooth muscle cells. Nat Biotechnol.

[17] Komada Y, Yamane T, Kadota D (2012). Origins and properties of dental, thymic, and bone marrow mesenchymal cells and their stem cells. PLoS One.

[18] Stanton LA, Underhill TM, Beier F (2003). MAP kinases in chondrocyte differentiation. Dev Biol.

[19] Prasadam I, van Gennip S, Friis T, Shi W, Crawford R, Xiao Y (2010). ERK-1/2 and p38 in the regulation of hypertrophic changes of normal articular cartilage chondrocytes induced by osteoarthritic subchondral osteoblasts. Arthritis Rheum.

[20] Zhao Y, Song T, Wang W (2012). P38 and ERK1/2 MAPKs act in opposition to regulate BMP9-induced osteogenic differentiation of mesenchymal progenitor cells. PLoS One.

[21] Twigg SR, Vorgia E, McGowan SJ (2013). Reduced dosage of ERF causes complex craniosynostosis in humans and mice and links ERK1/2 signaling to regulation of osteogenesis. Nat Genet.

[22] Nakamura T, Gulick J, Pratt R, Robbins J (2009). Noonan syndrome is associated with enhanced pERK activity, the repression of which can prevent craniofacial malformations. Proc Natl Acad Sci USA.

